# Three-dimensional colon cancer organoids model the response to CEA-CD3 T-cell engagers

**DOI:** 10.7150/thno.63359

**Published:** 2022-01-01

**Authors:** Alvaro Teijeira, Itziar Migueliz, Saray Garasa, Vaios Karanikas, Carlos Luri, Asunta Cirella, Irene Olivera, Marta Cañamero, Maite Alvarez, Maria C. Ochoa, Ana Rouzaut, Maria E. Rodriguez-Ruiz, Miguel F. Sanmamed, Christian Klein, Pablo Umaña, Mariano Ponz, Marina Bacac, Ignacio Melero

**Affiliations:** 1 Program of Immunology and Immunotherapy. Cima Universidad de Navarra. 31008. Pamplona, Spain.; 2 Centro de Investigación Biomédica en Red de Cáncer (CIBERONC), Madrid, Spain.; 3 Navarra Institute for Health Research (IDISNA), 31008, Pamplona, Spain.; 4 Departments of Oncology and Immunology. Clinica Universidad de Navarra, 31008, Pamplona, Spain.; 5 Roche Pharmaceutical Research & Early Development, Roche Innovation Center Zurich, Zurich, Switzerland.

**Keywords:** Colon organoids, T-cell engager, colon cancer, live confocal microscopy

## Abstract

**Rationale:** The CEA-CD3 T cell bispecific antibody cibisatamab (CEA-TCB) is currently undergoing clinical trials. Here we study its performance against three-dimensional tumor organoids in cocultures with T cells as compared to a higher affinity CEACAM5-CD3 (CEACAM5-TCB) bispecific antibody using time-lapse confocal microscopy.

**Methods:** Pre-labelled spheroids derived from colon cancer cell lines and primary organoids derived from four colorectal cancer surgical specimens, which expressed different graded levels of CEA, were exposed in cocultures to T lymphocytes. Cocultures were treated with CEA-CD3 T-cell engagers and were followed by live confocal microscopy. Caspase 3 activation detected in real-time was used as an indicator of tumor cell death. Co-cultures were also set up with autologous tumor-associated fibroblasts to test the co-stimulatory effect of a fibroblast activated protein (FAP)- targeted 4-1BBL bispecific antibody fusion protein currently undergoing clinical trials.

**Results:** Tumor-cell killing of 3D colon carcinoma cultures was dependent on the levels of surface CEA expression, in such a way that the lower affinity agent (CEA-TCB) did not mediate killing by human preactivated T cells below a certain CEA expression threshold, while the high affinity construct (CEACAM5-TCB) remained active on the low CEA expressing organoids. Modelling heterogeneity in the levels of CEA expression by coculturing CEA high and low organoids showed measurable but weak bystander killing. Cocultures of tumor organoids, autologous fibroblasts and T cells allowed to observe a costimulatory effect of anti-FAP-4-1BBL both to release IFNγ and to attain more efficacious tumor cell killing.

**Conclusion:** Three-dimensional tumor cocultures with T cells using live confocal microscopy provide suitable models to test the requirements for colon-cancer redirected killing as elicited by CEA-targeted T-cell engagers undergoing clinical trials and treatment allow combinations to be tested in a relevant preclinical system.

## Introduction

Bispecific antibodies binding a surface tumor antigen and CD3ε induce tumor redirected T cell killing 1. A bispecific T-cell engager BiTE redirecting killing to CD19 on B cells termed blinatumomab 2 has been approved for the treatment of acute lymphoblastic leukemia (ALL). The concept of T-cell redirection is being pursued for other malignant hematological diseases such as multiple myeloma targeting BCMA 3, 4. In essence, the agents bind to the tumor cells and only when attached to a plasma membrane, the monovalent anti CD3ε component triggers T-cell activation through the T-cell receptor leading to tumor cell killing 1.

In solid tumors, the concept has not been brought to fruition yet. This is in part because of the lack of good tumor specific surface moieties for targeting but efforts are being made targeting CEA 5-7 in colorectal cancer and PSMA for prostate cancer 8-10. In the case of CEA, two compounds have reached early-phase clinical trials. The first known as CEA-TCB (now termed cibisatamab) has intermediate/low affinity for a CEA epitope that is present only in the CEA form anchored to the plasma membrane 5. The second compound, termed CEACAM5-TCB binds with almost 200-fold higher affinity and to a more distal N-terminal epitope that is kept in the soluble released form. These agents had previously been tested preclinically to redirect T-cell killing against conventional tumor cell lines in culture and humanized immunodeficient mice co-engrafted with human tumor cell lines and CD34^+^ hematopoietic precursors 5, 11, 12.

Tumor spheroids and primary tumor organoids better reflect tumor biology as compared to cell lines growing in two dimensional cultures 13. In fact, patient- and mouse-derived organoids have been used to study tumor immunology and immunotherapy adding checkpoint inhibitors to such types of short-term cultures 14-17 and also in the study of resistance mechanisms to anti- CEA T cell engagers 18.

In this study, we use three-dimensionally cultured tumor cells in the presence of CEA redirected T-cell engagers to assess under live confocal microscopy the tumor killing performance in the presence of T lymphocytes. This model permits the testing of the influence of steroids that are prophylactically given in the clinic to mitigate the cytokine release syndrome, which is a common side effect of T-cell engagers 19. Moreover, the models can be improved to include FAP-expressing autologous fibroblasts 20 that mediate costimulation as provided by a FAP-targeted FAP-4-1BBL fusion protein, which is currently undergoing clinical development 11. 4-1BB (CD137) is a costimulatory receptor on T cells that is targeted in the clinic to elicit more powerful CD8-T cell-mediated antitumor immunity 21 and is exploited as a signaling component in second generation CAR T cells 22.

According to our work, primary tumor organoids provide a suitable model to study the performance and mechanistic requirements of T-cell engagers for solid tumors.

## Methods

### Tumor cell culture and spheroid formation

HT29 colon carcinoma tumor cells were obtained from ATCC and cultured in RPMI 1640 (Gibco) supplemented with 10% FCS under standard cell culture conditions (37 °C, 5% CO_2_). LS174T human colon carcinoma cells (ATCC CL 188), parental, modified to stably express red fluorescent protein (RFP) have been published elsewhere 12 and were cultured in DMEM (Gibco/Life Technologies) supplemented with 10% FCS, under standard cell culture conditions (37 °C, 5% CO_2_). LS180 (ATCC CL-187™) is a colorectal adenocarcinoma cell line. The cells were cultured in advanced DMEM with 10% FCS and 1% Glutamine and split every two to four days before reaching confluence. For spheroid generation 5,000 tumor cells were cultured in Matrigel (Corning) (8 mg/mL in PBS) domes of 25 μL for seven days before being used in cocultures. Y-27632 (Rho Kinase inhibitor) (Axon Medchem) was added to the culture media for the first three days of culture at a concentration of 10.5 mM.

### Human subjects

Organoids were generated from surgical specimens from four colorectal cancer patients that underwent surgery in the Clinica Universidad de Navarra. Written informed consent was obtained from all the patients and all procedures were approved by the Universidad de Navarra ethics committee (Study 2019.96). In some cases, blood was collected from the same patients under the same ethics protocol and informed consent, PBMCs were then isolated and frozen for later experiments.

Peripheral blood lymphocytes were obtained from the peripheral blood of a blood donor database mainly composed of graduate students (male and female, young adults) of the Universidad de Navarra (Pamplona, Spain) following written, signed, and dated informed consent according to a protocol approved by the institutional ethics committee (2019-76).

### Primary tumor organoid setup and culture

For isolation and establishment of primary tumor organoids, tumor was minced and incubated in DMEM-F12 Advance (Thermo), 5 mg/mL Collagenase II, 1.25 mg/mL Dispase, 2.5% FBS, 100× HEPES Buffer (Thermo), 100x Glutamax (Thermo), 100 ng/mL Primocin (Invivogen) on a rocker (From Miltenyi) at 37 °C for 2-3 h. The sample was collected in Advanced DMEM-F12, 10% FBS, 100x HEPES Buffer, 100x Glutamax, 100 ng/mL Primocin, passed through a cell strainer filter and centrifuged at 850 rpm for 7 min at 4 °C. Then 10 µL of 10 mg/mL DNase I were added and incubated at 37 °C for 5 min. Finally, the sample was resuspended in 100% Matrigel (8 mg/mL, Corning) and plated in a 24-wells plate which were pre-warmed and placed in the incubator at 37 °C for 30 min. When the Matrigel solidified, 500 µL of Complete Feeding Media (Advanced DMEM-F12, 100xHEPES Buffer, 100x Glutamax, 100 ng Primocin, 20% R-Spondin conditioned medium, 500 nM A83-01 (Sigma), 50 ng/mL hEGF (Preprotech), 100 ng/mL mNoggin (Preprotech), 100 ng/mL hFGF10 (Preprotech), 10 nM Gastrin I (TOCRIS), 3 μM SB202190 (Stem cell), 10 nM Prostaglandin E2 (TOCRIS), 1.25 mM N-acetylcysteine (Sigma), 10 mM Nicotinammide (Sigma), 1X B-27 Supplement (Thermo)) supplemented with 10.5 mM Y-27632 (Rho Kinase inhibitor) were added to the top of the Matrigel and the 24 well-plate placed in the incubator.

Organoids were passaged approximately every week. To split the primary tumor organoids, droplets were broken by adding 1mL of ice-cold Splitting Media (Advanced DMEM-F12, 100x HEPES Buffer, 100x Glutamax, 100 ng/mL Primocin) and pipetting up and down. Organoids were collected in a tube, centrifuged at 1200rpm for 5-10 min at 4 °C and the medium was removed. Organoids were broken up with a fire-polished pipette, centrifuged at 1200 rpm for 5-10 min at 4 °C. The pellet was mixed with Matrigel and plated in droplets of 50 μl each 24-platewell. After solidification, 500 µl Complete Feeding Media (DMEM-F12 Advance, 100x HEPES Buffer, 100x Glutamax, 125 μg/mL Primocin, 20% R-Spondin conditioned medium, 500 nM A83-01, 50 ng/mL hEGF, 100 ng/mL mNoggin, 100 ng/mL hFGF10, 10 nM Gastrin I, 3 μM SB202190, 10 nM ProstaglandinE2, 1.25 mM N-acetylcysteine, 10 mM Nicotinammide, 1X B-27 Supplement) was added.

Organoids were cryopreserved in Recovery Cell Culture Freezing Medium (ThermoFisher) as master and working biobanks. Organoids under passage 35 were used in experiments.

### T-cell isolation and culture

Total peripheral blood mononuclear cells (PBMCs) were isolated from peripheral blood by using a Ficoll gradient. Then they were plated in 10%FBS 1% P/E RPMI 1640 at a density of 2×10^6^ cells/mL with 1 μg/mL of plate bound anti CD3 (OKT3) and 5 mg/mL of soluble anti-CD28 antibodies for 48h. Then T cells were then cultured in new media containing 100 U/mL rhIL-2 (Proleukine) and 25 ng/mL rhIL-7 (immunotools) for an additional 8 days maintaining cells at a density of 10^6^ cells/mL and changing media every 2-4 days. In some cases, cultures were performed following the same protocol directly from frozen PBMCs.

### Isolation and culture of tumor associated fibroblasts

Tumor fragments of approximately 3x3x3 mm from the same specimens used to generate organoids were placed in 6 well plates in media containing 10% FBS 1% P/E RPMI 1640 for 7 days. After removal of the tumor pieces from the cultures the remaining attached cells corresponded to enlarged and adherent cells. 2D cultures were maintained for 5 passages until the establishment of a fibroblast culture and then surface FAP was measured by flow cytometry. Fibroblasts were cryopreserved in 10% FBS/DMSO as master and working biobanks. Fibroblasts under passage 10 were used in experiments.

### Tumor cell lysis of LS180 mediated by CEA- & CEACAM-TCB

Peripheral blood mononuclear cells (PBMCs) from a healthy donor (fresh in-house blood) were co-cultured with LS180 target cells in a ratio of 10 to 1. 25.000 target cells and 250.000 PBMCs were plated per well in flat-bottom 96-well plates and the respective TCB or media (untreated control) was added. The final concentration of the CEA-TCB & CEACAM-TCB ranged from 100 nM/20 nM to 6.4 pM/1.28 pM, respectively. Final volume per well was 200 µL. Plates were cultured for 48 h at 37 °C in a humidified incubator with 5% CO_2_. Target cell killing was assessed after 48 h of incubation by quantification of released lactate dehydrogenase (LDH) using an LDH detection kit (Roche Applied Science, Cat No.: 11644793001) according to manufacturer's instructions. As maximal release control, target cells were plated and 1% Triton X-100 (Bio Rad 161-0407-MSDS) was added 4 h before the LDH read-out. Absorbance was read on a Tecan Infinite F50. Raw absorbance data are shown as mean and standard deviation of triplicates.

### Video microscopy of co-cultures including tumor organoids/spheroids, T cells and fibroblasts activity assessments of bispecific antibodies

For the assessment of T cell mediated killing of tumor cells in cocultures with tumor spheroids or organoids and/or fibroblasts, tumor spheroids/organoids were recovered from the Matrigel using chilled PBS and gently pipetting. Spheroids/organoids were then stained with CMRA orange cell tracker dye (Thermo) for 20 min at 37 ºC in 10% FBS 1% P/E RPMI 1640. T lymphocytes and fibroblasts were stained following similar protocols using Cell tracker deep red and cell tracker green CMFDA respectively (in some experiments fibroblasts were stained with Cell tracker deep red). 150,000 T cells, the content of a 25 µL organoid containing dome (7 days of culture of 5,000 cells initiating the 3D cultures) (resulting in an approximate 1:5 tumor cell:T cell ratio) and 15,000 fibroblasts were placed in 5 µL of 15% Matrigel/15% collagen type I (Purecol, Ez Gel, Advanced Biomatrix) in the wells of a µ-Slide III 3D Perfusion plate (IBIDI). Chambers were mounted according to the manufacturer's instructions and the wells were filled with 250 µL of RPMI 1640 complete media (5%FBS, 1%P/E) containing the indicated drugs. The concentrations of the bispecific antibodies were as follows: 1 µg/mL of CEA-TCB, CEACAM5-TCB and mIgG_1_ (NA/LE, BD Pharmingen), 0.5 µg/mL of CEA-TCB and 10 µg/mL of (control, untargeted) (DP47-4-1BBL) and (Fibroblast activated protein targeted) FAP-4-1BBL unless indicated otherwise. Anti FAS-L (Rand D) and anti-IFNγ (RandD) blocking antibodies were used at a concentration of 10 and 4 µg/mL respectively, according to manufacturers' instructions.

Cultures were maintained for 2-5 hours at 37 ºC to stabilize the co-cultures before adding 5 µM a Caspase 3 activity fluorescent probe (Incucyte^®^ Caspase-3/7 Green Dye for Apoptosis; Essen Bioscience, Sartorius) that once is cleaved by active Caspase 3 interacts with nuclear DNA and generates green fluorescence.

Time lapse videos were recorded in an LSM880 inverted confocal microscope (Zeiss) equipped with an incubator to maintain 5% CO_2_, humidity and a temperature of 37 ºC of temperature throughout the experiment. Images were taken using a 40× water plan apochromat LD objective (NA 1. 2) except for the videos in figures 4 and 5 in which fibroblasts were present in which case acquisition was performed with a 25× LD water immersion objective (NA 0.8). An Argon 488 laser cell line and two He/Ne lasers (543 and 633 nm) were used to simultaneously excite the three dyes. 512x512 pixels 3D Z stacks of 60-80 micrometers were obtained by acquiring images every 1.6 µm. Z- stacks were taken every 3 minutes for up to 12 hours. Then plates were removed from the microscope, set up in an incubator at 37 ºC, 5% CO_2_ until a new video was started for the same plate at a time point 26 h after the setup of the coculture. In some cases, (Videos on figure 3 and 5) time-lapse videos were recorded over longer periods of time as indicated in individual videos or quantifications.

### Flow cytometry

For assessment of spheroid/organoid cocultures by flow cytometry, cultures were set up with 350,000 T cells, the content of one 25 µL organoid containing domes (7 days of culture of 5,000 cells initiating the 3D cultures) and the indicated amount of fibroblasts were placed in 15% Matrigel/15% collagen type I in 48 well flat bottom culture plates with 1 µg/mL of CEA-TCB, CEACAM5-TCB and mIgG_1_ (NA/LE, BD Pharmingen) unless indicated otherwise. Co-cultures were recovered at the indicated time points with chilled PBS and dissociated to single cells using TrypLE Express (Thermo).

Individual cell suspensions were stained with the following antibodies: anti-human CD45-PB, anti-human CD8 BV510 and Alexa 488, anti-human CD4 FITC and PercP-Cy5.5, anti-human CD137 PE, anti-human Epcam PercP Cy5.5, anti-human CEA APC, anti-human FAP Alexa Fluor 647 all from Biolegend), anti-human Caspase 3 Alexa Fluor 647 (BD Pharmingen). Dead cells were stained using Zombie Nir death cell infrared dye (Biolegend) and to stain for Caspase 3 cells were previously treated with Cytofix/Cytoperm kit (BD) according to manufacturer's instructions. Flow cytometry was performed using a Cytoflex S cell cytometer (Beckman Coulter). Data was analyzed using FlowJo software (BD).

### Interferon-γ ELISA

To study interferon gamma production in cocultures with T cells, fibroblasts and organoids triple co-cultures were set following the same protocol for flow cytometry. Co-stimulation was provided by treating with FAP targeted 4-1BBL constructs or appropriate controls. Cultures were treated with 0.5 µg/mL CEA TCB or mIgG_1_ and 10 µg/mL FAP-4-1BBL or UT-4-1BBL (DP47-4-1BBL) for 72 hours. Supernatants were recovered from the cultures by diluting in 1 volume of PBS the content of the wells following centrifugation at 3000 r.pm. Then the amount of IFN gamma in these supernatants was measured with a Human IFN-γ ELISA Set (BD) according to manufacturer's instructions.

### CEA Immunostaining of FFPE biopsies from colorectal tumor samples

FFPE biopsies from colorectal tumor samples were sectioned to a four-micrometer thickness, deparaffinized and stained for the IVD mouse monoclonal antibody against CEA (clone CEA31, Roche) on VENTANA BenchMark ULTRA instrument and detected using DAB chromogen.

### Image analysis

To perform caspase 3 activation analysis in time-lapse microscopy videos, Imaris 7 software was used (Bitplane). Individual organoid volumes were calculated at time point 0 using the surface tool. Caspase 3-positive events were also segmented by using the volume tool in the green fluorescent channel. Surface detail was setup to 1 µm and particle size was set at 8 µm. Image segmentation was performed at the indicated timepoints and caspase 3-positive T cells and green fluorescence not present within tumor cells were excluded from the analysis. In videos where CMFDA stained fibroblasts (green fluorescent) were present, such cells were excluded from the analyses based on morphology (full fibroblast shape in contrast to the nuclear staining that features the caspase 3 probe).

Representative videos and images were generated using IMARIS or Zen Black (Zeiss) software and then edited with Final Cut Pro (Apple inc) software.

### Statistical analysis

All data were analyzed using Graph Pad Prism 8. Means and standard deviations of the means are presented as averages and error bars as indicated in the figure legends. Mann-Whitey's U tests or Student's *t* tests were used to analyze statistical differences between groups unless otherwise indicated. *p* values are shown for any relevant statistical difference in the figures.

## Results

### Differential redirected killing of colon cancer spheroids by CEA-T-cell engagers depends on the level of surface CEA expression

Two different CEA-CD3ε T cell bispecific antibodies were generated 5. As shown in Figure S1 they have a similar structure and contain the same monovalent CD3 engaging domain. However, their bivalent CEA-binding parts bind with ten-fold different affinity and to distinct epitopes of the CEA molecule. The lower affinity agent is termed CEA-TCB (cibisatamab), while the higher affinity agent is referred to as CEACAM5-TCB. In 2D cultures of colon cancer cell lines in the presence of human T cells the two constructs showed a ten-fold difference in the EC_50_ for tumor cell killing 5 (Figure S1B).

We used two well described colon carcinoma cell lines with different levels of surface CEA to set up spheroids that grew in culture as three-dimensional structures.

**Figure 1:**
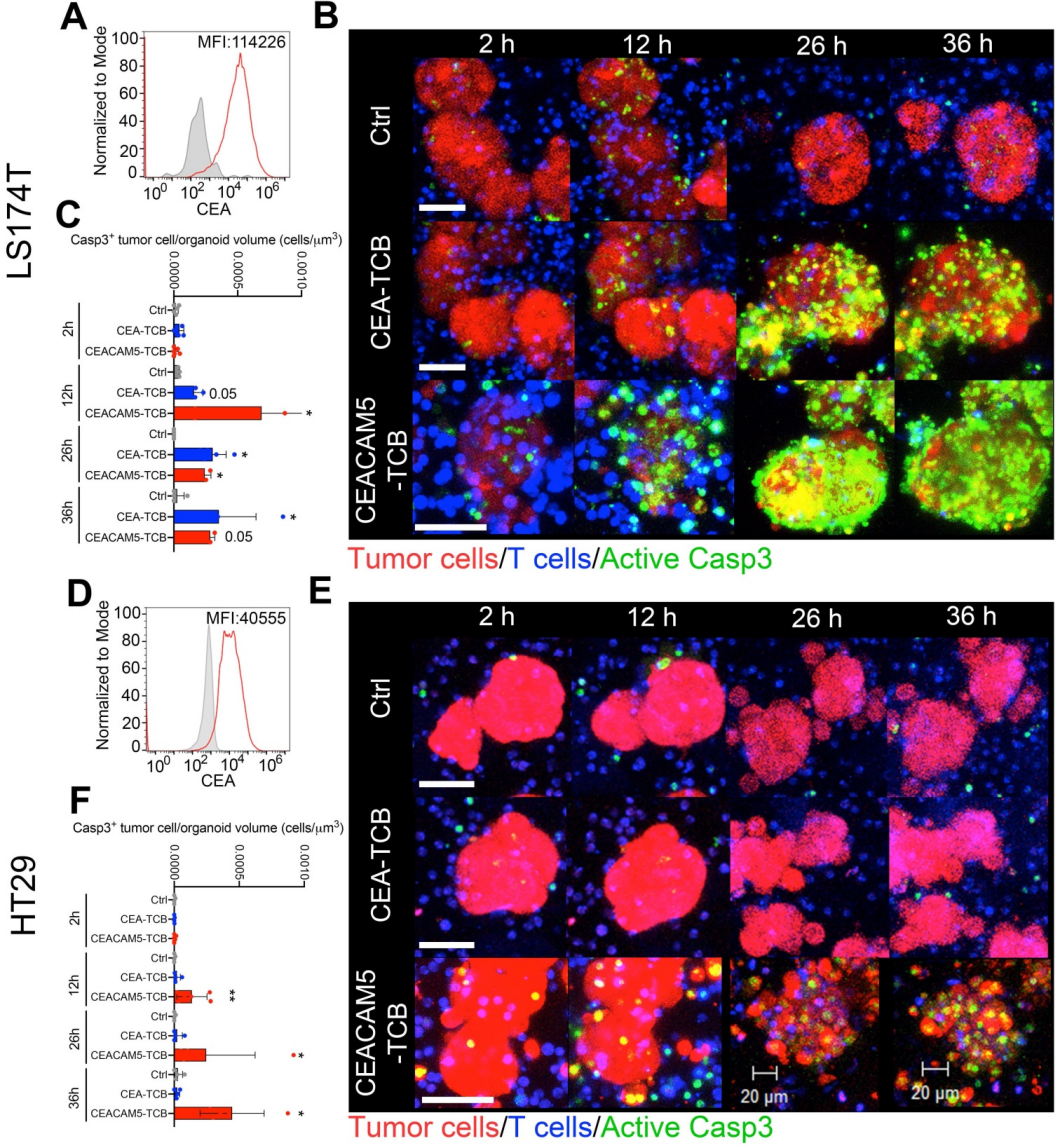
** Modelling cytotoxicity of CD3-CEA bispecific T-cell engagers of different affinity on 3D tumor spheroids. (A)** Representative flow cytometry histograms of CEA surface expression on LS174T cells. **(B)** Representative images of cocultures of LS174T spheroids (Red) with T cells (Blue) and the CEA-TCB, CEACAM5-TCB T-cell engagers and an irrelevant mouse IgG_1_ control (Ctrl). Active caspase 3 is shown by a fluorescent probe (Green) that marks apoptotic tumor cells (Bars 50 μm). **(C)** Quantification of apoptotic cells in images as in B and videos as Video S1. **(D)** Representative flow cytometry histogram of CEA surface expression on HT29 cells. **(E)** Representative images of cocultures of HT29 spheroids (Red) with T cells (Blue) and the CEA-TCB, CEACAM5-TCB T-cell engagers and an irrelevant mouse IgG control (Ctrl). Active caspase 3 is shown by a fluorescent probe (Green) that indicates apoptotic tumor cells (Bars 50, 20 μm). **(F)** Quantification of apoptotic cells in images as in E and videos as Video S1. Representative experiments are shown out of at least two rendering similar results. Quantifications in C and F were performed analyzing four to seven spheroids per condition. Means±SD are shown for quantitative data. * p<0.05; U Mann Whitney tests were performed for statistical comparisons.

The spheroids derived from the LS174T cell line which expresses high levels of surface CEA (Figure 1A) were readily killed by preactivated T cells when treated with either CEA-TCB and CEACAM5-TCB at sufficient concentrations. This was assessed in time-lapse confocal microscopy videos in which T cells and tumor spheroids were prelabelled and apoptosis was visualized in real time by a fluorescent probe detecting apoptotic cells containing active cleaved caspase 3 (Figure 1B and Video S1). Quantification of death-cell volume in the organoids is provided in Figure 1C.

By striking contrast, when spheroids derived from the HT29 cell line that expressed markedly lower levels of surface CEA (Figure 1D) were used in the same way, the killing of the spheroids was lost for CEA-TCB at saturating concentrations 5, but was preserved for CEACAM-5 TCB (Figure 1E-F and Video S1).

The killing in such experiments was exerted by preactivated and expanded human peripheral blood T cells pre-activated with plate-bound anti CD3 and CD28 antibodies in the presence of IL-2 and IL7 for 15 days. Such cultured lymphocytes did not significantly modify the relative percentage of CD4 and CD8 T cells present in peripheral blood (Figure S2A). Of note, killing of LS174T spheroids was also achieved by freshly isolated T lymphocytes (Figure S2B) and total peripheral blood mononuclear cells (Figure S2C).

### Levels of CEA surface expression on primary tumor organoids determine efficient redirection of cytotoxicity by T-cell engagers

We derived four organoids from fresh surgical specimens from colorectal cancer patients. They were selected to represent different levels of CEA expression.

**Figure 2:**
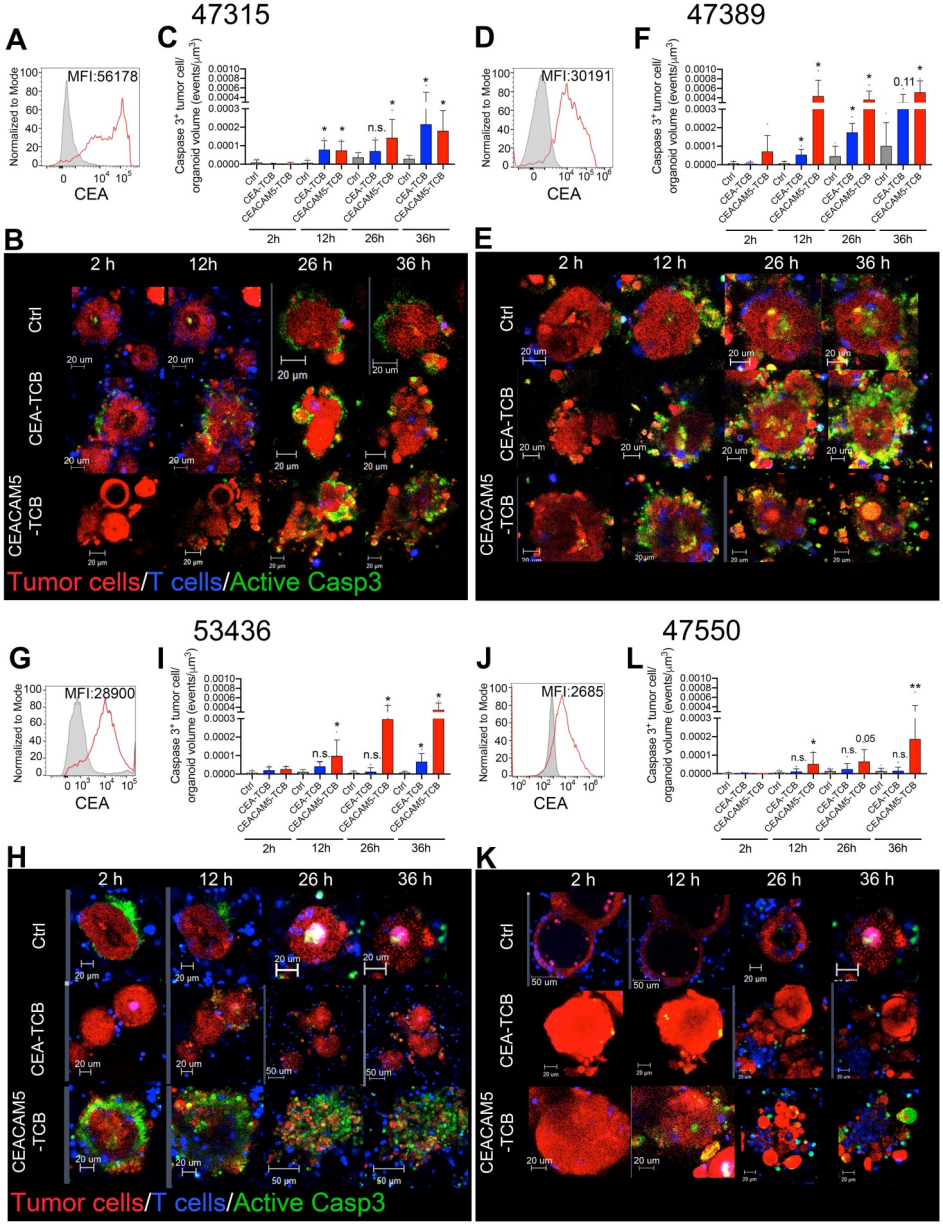
** Modelling T-cell engager-induced cytotoxicity on primary colon cancer organoids.** Organoids (Red) generated from surgical specimens from four different colon cancer patients were cocultured with T cells (Blue) and the CEA-TCB, CEACAM5-TCB T-cell engagers and an irrelevant mouse IgG control (Ctrl). Active caspase 3 is shown by a fluorescent probe (Green) that indicates apoptotic tumor cells. Panels **A, D, G and J** show representative histograms of CEA surface expression of such four organoids as measured by flow cytometry. **B, C, H and K** show representative confocal microscopy snapshots of time-lapse videos of cocultures of the four different organoids treated with bispecific T cell engagers. Panels **C, F, I and L** show quantitative data of caspase 3 activation as in Video S2 at different time points. Data come from at least two experimental replicates rendering similar results. Quantifications were performed analyzing four to eight organoids per condition. Means±SD are shown for quantitative data. * p<0.05; U Mann Whitney tests were performed for statistical comparisons.

Organoids 47315 and 47389 showed high and intermediate-high levels of CEA surface staining when single cell suspensions were analyzed by flow cytometry (Figure 2A, D). In these cases, cocultures with T cells imaged as in Figure 1 showed prominent killing both by CEA-TCB and CEACAM5-TCB (Representative images and quantitative data shown in Figure 2A-F). These cocultures were performed in RPMI 10% FBS media to ensure proper activation of T cells (as T cells showed impaired activation in colon cancer organoid feeding media) (Figure S3A). Culture of organoids in RPMI 10% FBS had a minimal impact on organoid viability and architecture during such short-term cultures (Figure S3B). Yet some minor baseline apoptosis could be observed in control organoid/T cell co-cultures overtime even in the absence of CEA-TCBs.

The 53436-organoid showed intermediate-low levels of CEA and poor sensitivity to killing by CEA-TCB, while preservation of killing by CEACAM5-TCB (Figure 2G-I). In the case of organoid 47550 with detectable but lower CEA expression (Figure 2J), redirected killing was only observed with CEACAM5-TCB but not with CEA-TCB (Figure 2L, K). A representative time-lapse recording of organoids in Figure 2 is shown in Video S2.

To confirm these results with autologous preactivated T cells, we performed the experiment in Figure S4 in a case in which the patient from whom the organoid was derived donated peripheral blood lymphocytes. These experiments showed comparable killing results as those shown in Figure 2 in the case of organoid 47389 with high levels of CEA expression.

### Weak bystander killing effects by CEA T-cell engagers

**Figure 3:**
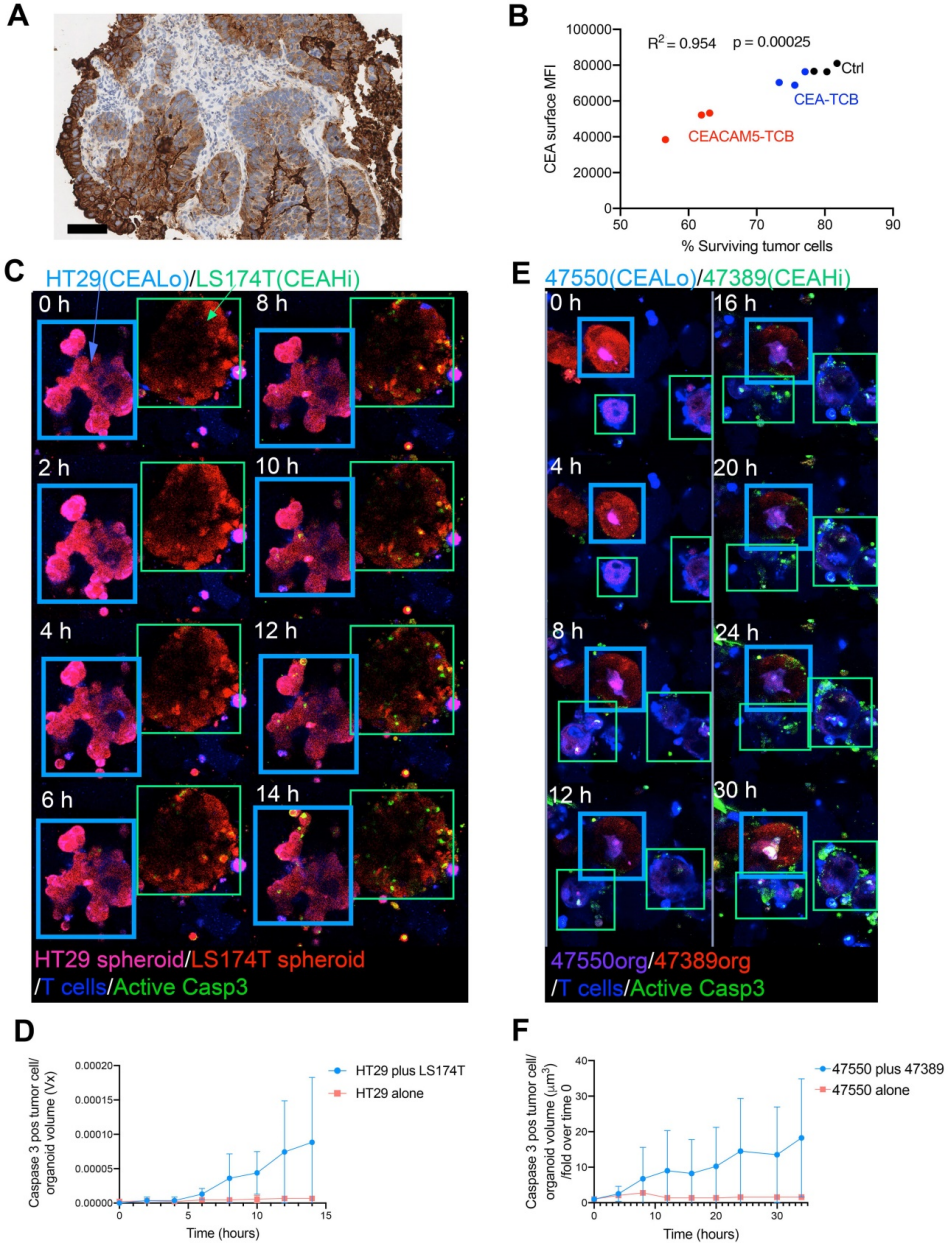
** Limited bystander killing mediated by T-cell engagers. (A)** Representative immunohistochemistry of CEA expression in a case of colorectal cancer showing wide heterogeneity in CEA intensity in different regions. **(B)** Cocultures of T cells with the organoid 53436 expressing medium levels of CEA were treated with CEA-TCB, CEACAM5-TCB or irrelevant control (Ctrl) and recovered after 24 hours. Relative numbers of tumor cells in the cocultures and surface CEA levels were then assessed then by flow cytometry. Correlation of surface CEA expression with the percentage of tumor cells in culture as a surrogate marker for T cell-mediated killing. **(C,D)** Cocultures of HT29 (CEA low spheroids, pink) and LS174T (CEA high spheroids, Red) were setup with T cells (Blue) and a caspase 3 active fluorescent detection probe (Green) to follow tumor cell killing over time. In another well the same cocultures were placed using HT29 cells only. Both conditions were treated with CEA-TCB T-cell engagers and followed overtime by time-lapse confocal microscopy. (C) Representative snapshots showing such cocultures. (D) Quantification of videos as in C when compared to cocultures of HT29 spheroids alone performed in parallel. **(E,F)** A similar experiment to the one using spheroids in C and D was performed using CEA^High^ organoids (Patient 47389 (Purple) and CEA^Low^ organoids (Patient 47550 (Red)). (E) Representative snapshots showing such cocultures over time. (F) Quantification of videos as in C in comparison with cocultures of 47550 organoids co-cultured with T cells alone performed in parallel. Means±SD are shown for quantitative data.

Colon cancer usually shows considerable levels of spatial heterogeneity regarding CEA surface expression as shown in a representative case by IHC in Figure 3C and as also previously shown by others 18. Exposure to CEA T-cell engagers might, in the presence of T cells, result in efficient killing of high-CEA expressing tumor cells and a subsequent selection of cells expressing low CEA levels. Indeed, organoids co-cultured with T cells for 24 hours in the presence of CEACAM5-TCB and CEA-TCB showed reduced levels of CEA expression on the remaining tumor cells, which were clearly fewer and with more reduced CEA expression in the case of the more target-avid CEACAM5-TCB (Figure 3B).

Accordingly, it would be of great therapeutic interest if tumor cell redirected cytotoxicity could be amplified by bystander killing of these cells with lost or reduced CEA expression levels. To model this situation in 3D cultures, we mixed differently prelabeled spheroids from HT29 and LS174T and used confocal microscopy to monitor killing events on both types of spheroids in the presence of CEA-TCB and T-cells. We compared killing events when only insensitive HT29-derived spheroids were present or when cocultured with sensitive LS174T. As quantified in Figure 3D, killing events were increased in the co-cultured spheroids but only to a weak extent in terms of spheroid volume (Video S3).

Similarly, we performed experiments coculturing CEA^high^ and CEA^low^ primary tumor organoids. Again, time lapse confocal microscopy detecting tumor cells undergoing caspase 3 activation provided evidence for some degree of bystander killing. Accordingly, death events were more frequent among the CEA^low^ organoid 47550 when co-cultured with the CEA^high^ organoid 47389. Quantitative data are presented in figure 3F, that demonstrates that there is some level of bystander killing but of limited, non-eradicative intensity (Video S3).

**Figure 4:**
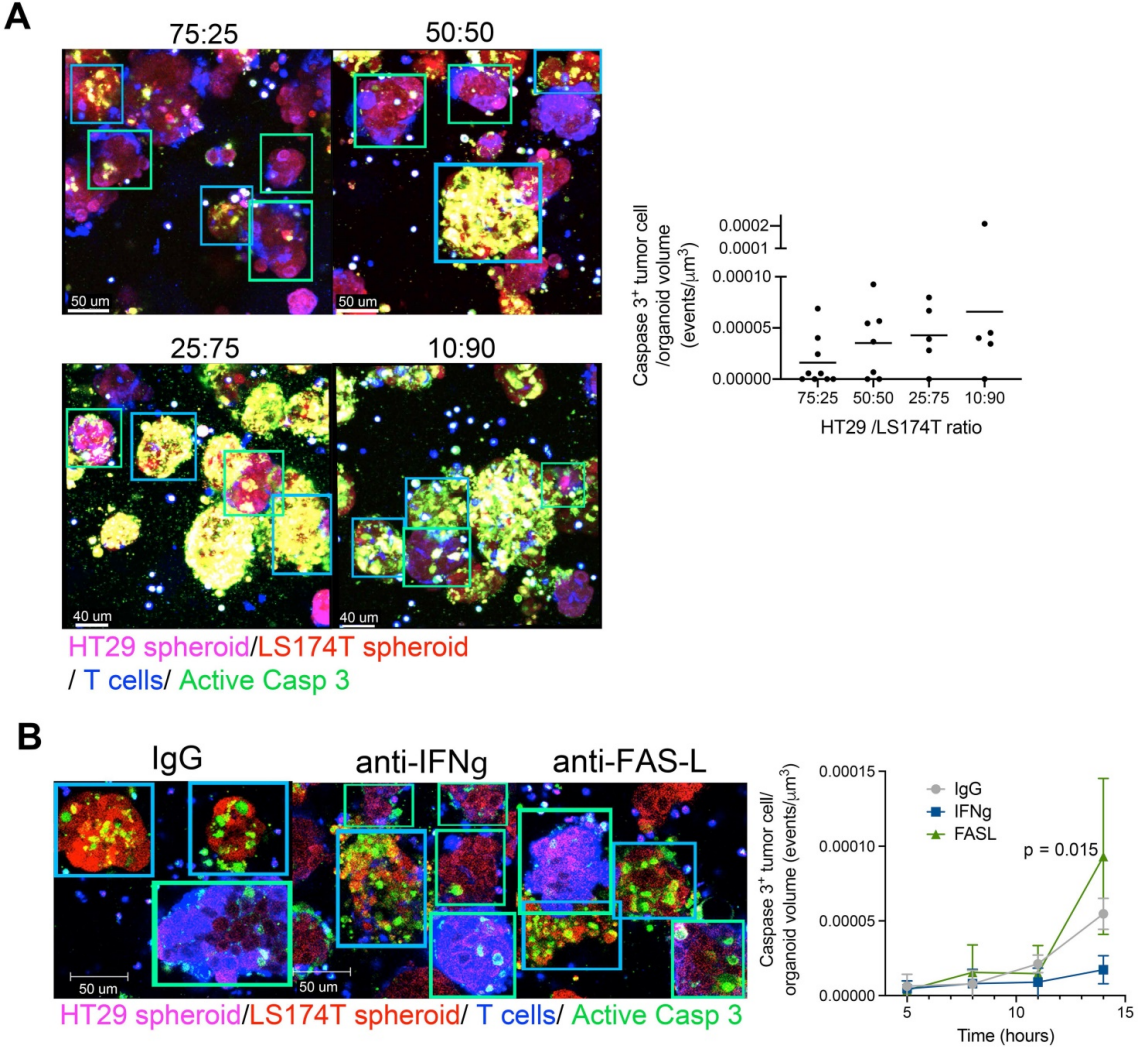
** Bystander killing mediated by CEA targeted T-cell engagers is increased when augmenting the proportion of CEA^HI^ tumor cells and is dependent on INFγ. (A)** Cocultures of HT29 (CEA low spheroids, pink) and LS174T (CEA high spheroids, Red) were setup with T cells (Blue) and a caspase 3 active fluorescent detection probe (Green) at increasing LS174T-spheroid to HT29-spheroid ratios. All conditions were treated with CEA-TCB T-cell engagers and followed overtime by time-lapse confocal microscopy. Representative snapshots showing such cocultures and a quantification of the videos at the 16 hours' time point are shown. **(B)** Cocultures of HT29 (CEA^Low^ spheroids, pink) and LS174T (CEA^High^ spheroids, Red) (50:50 ratio) were setup with T cells and treated with CEA-TCB (1 μg/mL) and anti FAS-L (10 μg/mL), anti IFNγ (4 μg/mL) or an irrelevant IgG control (10 μg/mL). Representative snapshots showing such cocultures at the end of the videos (14 hours) and a quantification of caspase 3 activity induction overtime are shown. Means±SD are shown for quantitative data. Statistical comparisons were performed with U Mann Whitney tests.

Bystander killing of HT29 CEA^low^ spheroids was increased when the proportions of CEA^Hi^ spheroids in the cocultures were increased, suggesting that the bystander effect was mediated by T-cell-engager-mediated T cell activation (Figure 4A). Mechanistically, the observed bystander effect required IFNγ secretion but not FAS-L (Figure 4B).

Another clinically relevant question is whether steroids attenuate the redirected T-cell killing effect. In figure S5A, B and C we show that surviving tumor cells in co-cultures with spheroids from LS174T cell line and tumor killing events were not reduced if dexamethasone was present in the culture media. Moreover, dexamethasone had no impact on CD4 and CD8 T-cell activation as measured by CD137 induced expression (Figure S5B, E and F). The relevance of these findings comes from the fact that steroids are usually given to patients as a medication to mitigate cytokine release syndrome secondary to these and other T-cell engagers.

### Cocultures of organoids and autologous tumor-associated fibroblasts allow testing costimulation by a FAP-targeted 4-1BBL construct

To costimulate T cells in tumors a construct targeting FAP has been published 11. FAP-4-1BBL is an antibody encoding a trimeric 4-1BBL recombinant protein in one arm of the construct and a FAP binding antibody in the other arm (Figure S1A). Such a moiety binds to FAP and can crosslink, and thereby activate, CD137 (4-1BB) on the T cells that would come into contact with fibroblasts while undergoing activation.

**Figure 5:**
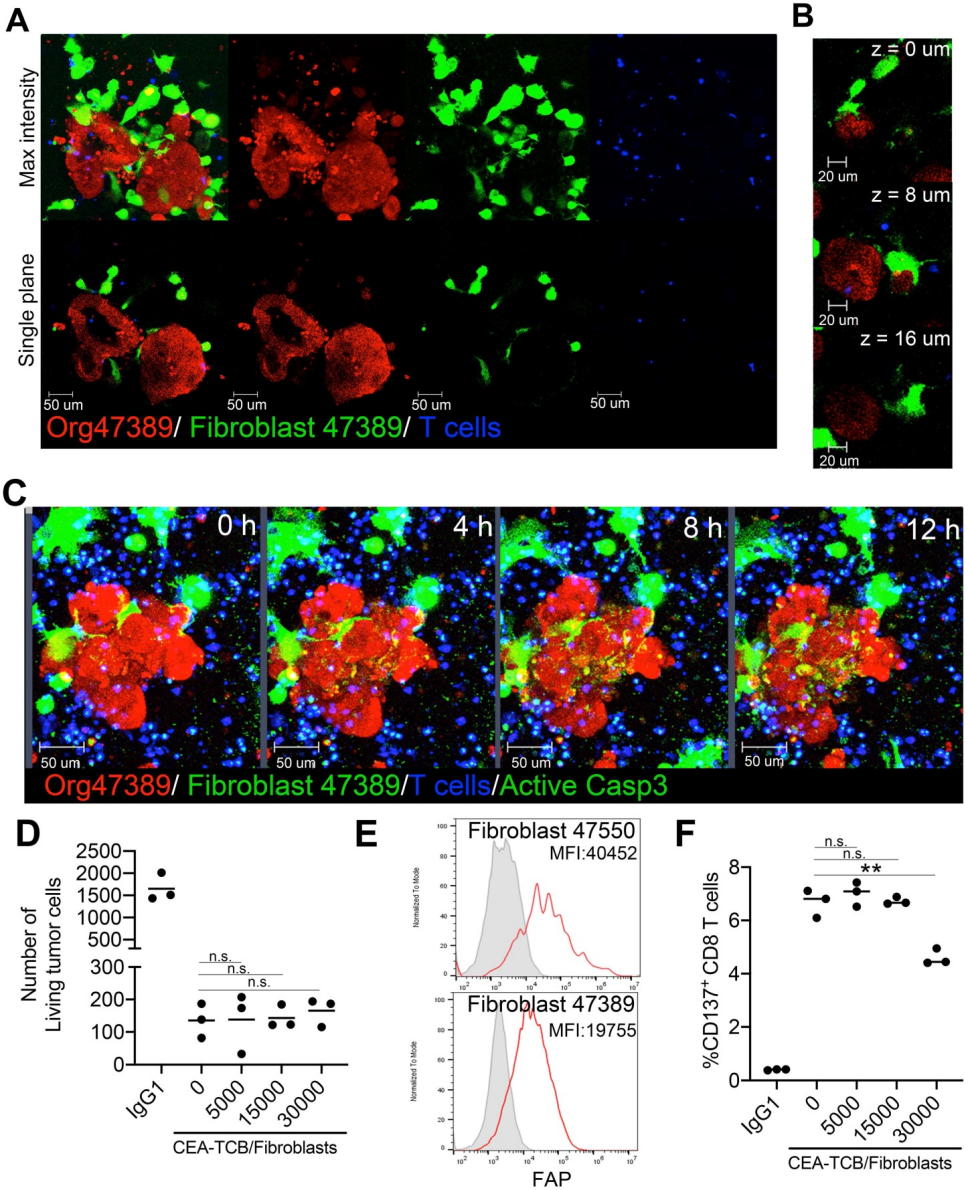
** Co-cultures of tumor organoids with autologous FAP^+^ fibroblasts do not impair CEA redirected killing by T cells. (A)** Representative images of a coculture of tumor cell organoids (Red) and tumor-associated fibroblasts (Green) from the patient 47389 with allogeneic T cells. A 3D reconstruction and a single plane of the same two organoids are shown to better visualize the interactions of tumor cells with fibroblasts. **(B)** Series of Z-stack planes of an organoid/fibroblast/T-cell co-culture as in A to show close contact of fibroblasts with organoids. **(C)** Time-lapse sequence of a triple Organoid/fibroblast/T-cell coculture treated with CEA-TCB antibodies and showing tumor cell cytotoxicity by activated caspase 3. **(D)** LS174T spheroids were set up in cocultures with T cells and CEA-TCB antibody or IgG_1_ irrelevant control antibody (Ctrl) and different amounts of fibroblasts (0,5x10^3^, 1.5x10^4^, 3x10^4^ fibroblasts) were added to the culture. Numbers of recovered tumor cells assessed by flow cytometry after 24 hours of coculture are shown. **(E)** Representative flow cytometry histogram of fibroblast activated protein (FAP) expression on fibroblast in cultures from patients 47389 and 47550. **(F)** CD137 induction on CD8 T cells in cocultures as in D as measured by flow cytometry.

First, we plated co-cultures of the CEA^high^ organoid 47389 with autologous tumor-associated fibroblasts that were labelled in green. Inclusion of fibroblasts in the cocultures favored tumor organoid survival even in RPMI 10% FCS media (Figure S3B). As shown in Figure 5A and B, such co-cultures were feasible and as shown in figure 5C, T cells could be added and visualized in such cultures (Video S4). In this setting, CEA-TCB can redirect killing that was not affected by the presence of fibroblasts as shown in Figure 5C and D. Autologous fibroblasts cultures from two of the tumors from which organoid cultures had been derived showed clearly detectable levels of surface fibroblast associated protein (FAP) that could be the target of FAP-targeted therapeutic constructs (Figure 5E). Of note, T cells during the cocultures expressed surface CD137 as a result of activation upon CEA-redirected killing encounters (Figure 5F).

**Figure 6:**
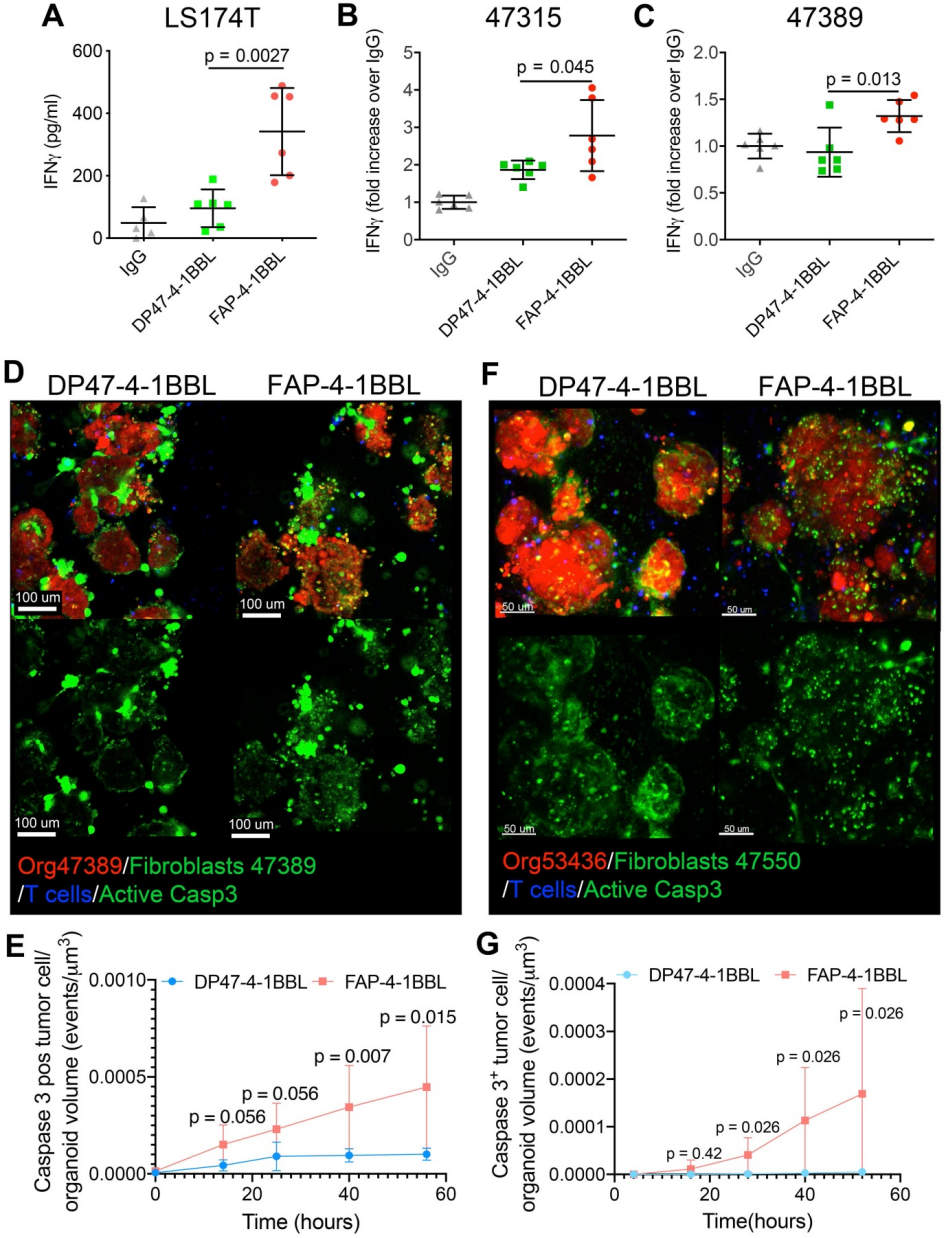
** Costimulation of CEA T-cell engagers by a FAP targeted 4-1BB ligand. (A,B,C)** Spheroids from LS174T cell line (A) or organoids from patients 47315 (B) and 47389 (C) were set up in cocultures with tumor associated fibroblasts and T cells and to be treated with irrelevant mIgG_1_ antibody, CEA-TCB plus untargeted control 4-1BBL (DP47-4-1BBL) or FAP targeted 4-1-BBL (FAP-4-1BBL). 72 hours after the coculture setup, IFNγ in the supernatants was measured by ELISA. Results come from two pooled experiments with triplicate wells per condition. **(D and F)** Representative microscopy images of cocultures of tumor organoids 47389 (D) and 52436 (F) (Red), autologous fibroblasts (Green) and T cells (blue) treated with CEA-TCB plus DP47-4-1BBL or FAP-4-1BBL following 48 hours of coculture. Induction of apoptosis in tumor cells is shown by the caspase 3 activity probe also in green. The green channel of the images is shown apart to better identify caspase 3 positive cells and differentiate them from green fibroblasts. **(E and G)** Quantitative assessment of caspase 3 activity induction on tumor organoids overtime as in Video S5 showing fibroblast/organoid/T-cell co-cultures treated with CEATCB plus UT or FAP 4-1BBL using 47389 CEA^High^ organoids (E) and 53436 CEA^Low^ organoids (F). D to G show representative data from three and two experiments rendering similar results. Means±SD are shown for quantitative data. U Mann-Whitney tests were used to assess for individual significant differences between the relevant conditions.

Taking advantage these conditions, the FAP-4-1BBL construct (Figure S1A) was tested. As a control an untargeted 4-1BBL construct that is not crosslinked by FAP was used at equal concentrations. Figures 6A-C show that FAP-4-1BBL but not untargeted 4-1BBL costimulated IFNγ secretion to the supernatant by redirected T cells with CEA-TCB. This was observed in the LS174T spheroids and in the two primary tumor organoids tested when co-cultured in the presence of autologous FAP^+^ fibroblasts.

Importantly, confocal videomicroscopy showed increases in killing events in the 47389 organoids, in the presence of FAP-4-1BBL and its crosslinking by fibroblasts (Figure 6D and E, Video S5 and Figure S6). Such increased cytotoxicity induced by FAP-4-1BBL was also observed when treating the CEA^low^ colon cancer organoid (53436) that did not experience cytotoxicity in the presence of the CEA-TCB T cell engager alone (Figure 6F and G and Figure S6). These results indicate that the expected synergy between CEA-TCB and FAP-4-1BBL could be recapitulated in 3D tumor coculture systems encompassing tumor organoids, T cells and autologous fibroblasts.

## Discussion

T-cell engagers that redirect T-cell mediated cytotoxicity to tumor cells offer many advantages and opportunities 1. They mimic strong antigen recognition on tumor cells resulting in a strong “signal one” as delivered by the TCR-CD3 complex 1. Success in hematological malignancies has several causes: (i) contacts between malignant cells and T cells are probably not limited; (ii) targeted malignant B cells often co-express costimulatory ligands; (iii) targets such as CD19, CD20 or BCMA are lineage-specific and not found on other cell types and are expressed homogenously and at bright levels. In solid malignancies, however, tumor-selective surface targets are difficult to find, and most antigens are also expressed at low levels on non-transformed epithelial tissues. Furthermore, heterogeneity and levels of target expression constitute an important issue and contacts of malignant and T cells are contingent on the baseline levels of CD4 and CD8 T-cell infiltration in the tumor microenvironment 23. In the current scenario, reliable experimental models to study the treatment limitations, so as to refine the agents to better understand their mechanisms and to test all combinations constitute major goals in themselves.

With the help of time-lapse video microscopy, we have monitored plated cocultures of tumor cells forming three dimensional structures with preactivated T lymphocytes and autologous tumor-associated fibroblasts. Further sophistication of the cocultures will be attempted to recapitulate the myeloid leukocyte compartment 24. Even if imperfect, the model visualized tumor cell apoptotic demise in real-time, as revealed by caspase 3 activation as executed by T cells redirected under the influence of two T-cell engagers targeting CEA.

By taking advantage of this novel technology, important aspects have been revealed. First, CEA expression levels dictate the susceptibility of the system to meaningful T-cell killing. Critical threshold levels of CEA expression are specific for each T cell engager, depending on the binding affinity for the CEA target but not on the affinity for CD3ε. We were therefore able to confirm results observed in 2D cultures in which CEA levels also dictated the efficacy of CEA-TCB-induced killing of tumor cells 5. Second, when susceptible and CEA-TCB sensitive organoids/spheroids are co-cultured, some level of bystander killing of the low CEA expressing tumors is found, but to a level that would very doubtfully be clinically useful. This bystander killing was required INFγ secretion as previously shown in other solid tumor models 25, 26. FAS-L, reportedly a mediator of bystander cytotoxicity 27 was not required for bystander killing in our experimental system. Yet, such low bystander killing effect may be more clinically relevant for other TCBs, such as CD20-T cell-engagers in which bystander killing may prevent outgrowth of CD20 low or negative clones. Of important note, in the culture systems T cells are plated in excess and would mimic a hot tumor densely infiltrated by CD8 and CD4 T cells 28, 29. Previous work on colon cancer organoids cultured with T cells had shown the importance of homogenous and intense levels of CEA expression for cancer elimination by cibisatamab. Recent *in vivo* experimental evidence supports this notion, highlighting that high antigen expression is a necessary condition for the efficacy of T cell engagers 30. Accordingly, colon cancer patients are prescreened for high mRNA expression of CEA before entering ongoing clinical trials 18.

All these aspects together with the fact that steroids do not affect redirected cytotoxicity allow important clinically relevant conclusions to be drawn: (i) low-affinity T-cell engagers may more strongly depend on the level of target antigen expression, (ii) treatment may convey inflammatory cytokine production and local inflammation. The solution of engineering higher affinity for CEA has the ensuing danger of the weak CEA positivity on the non-malignant cells lining the gut 31. Nonetheless, even if the higher affinity version is more effective, it could also be blighted by heterogenous levels of expression of the CEA targets. Although we did not evaluate possible cytotoxic effects of T cell engagers in non-transformed colon organoids the high response engaged by the high affinity CEACAM5-TCB towards organoids expressing very low levels of CEA indicates that the potential toxicity of such a high affinity construct may be a major issue in its clinical development.

Very interestingly, another bispecific construct has been generated to convey 4-1BB co-stimulation to areas in which fibroblasts are expressing FAP 11, that is restricted to tumor- associated fibroblasts and those involved in wound healing 32, 33. The FAP-4-1BBL construct co-stimulated T cells offering costimulatory “signal 2” in the cocultures of tumor organoids, T cells and fibroblasts. 4-1BB costimulation concomitant to CD3 engagement reproduces the two signals introduced in second generation CAR T cells 34, 35. In our hands such costimulatory activity was detectable and functionally relevant, even in CEA low tumor organoids. Indeed, these effects and previously reported results in xenograft humanized mouse models 11 are supportive of a combination clinical trial of cibisitamab (CEA-TCB) and FAP-4-1BBL (NCT04826003). The signal 1 and 2 is often useful when considering immunotherapy combinations (2620540, 24485523).

Our experimental system permits real-time visualization and allows for improvements to better recapitulate the tumor tissue microenvironment and the performance of T cells. Importantly, this setup allows other immune combinations which might be pursued in the clinical setting to be tested. Three dimensional structures, cocultures of multiple stromal cellular and matrix components, modeling paucity of T cells and other factors such as oxygen and nutrient deprivation 36, 37 will be amenable to be modelled in the near future using similar experimental systems.

Even if not subjected to further improvements, the models are especially suitable to study T-cell-engagers and their anti-tumor activity, as shown for these two T-cell engagers.

## Supplementary Material

Supplementary figures and movie legends.

Supplementary movie 1.

Supplementary movie 2.

Supplementary movie 3.

Supplementary movie 4.

Supplementary movie 5.
