# Supplementary material for: Three-dimensional colon cancer organoids model the response to CEA-CD3 T-cell engagers
Source: Theranostics. 2022 Jan 1;12(3):1373–87. doi: 10.7150/thno.63359 (PMC8771540; doi:10.7150/thno.63359)
Supplement: Supplementary file 1 — Supplementary figures and movie legends. [file thnov12p1373s1.pdf]

Supporting Information

Figure S1

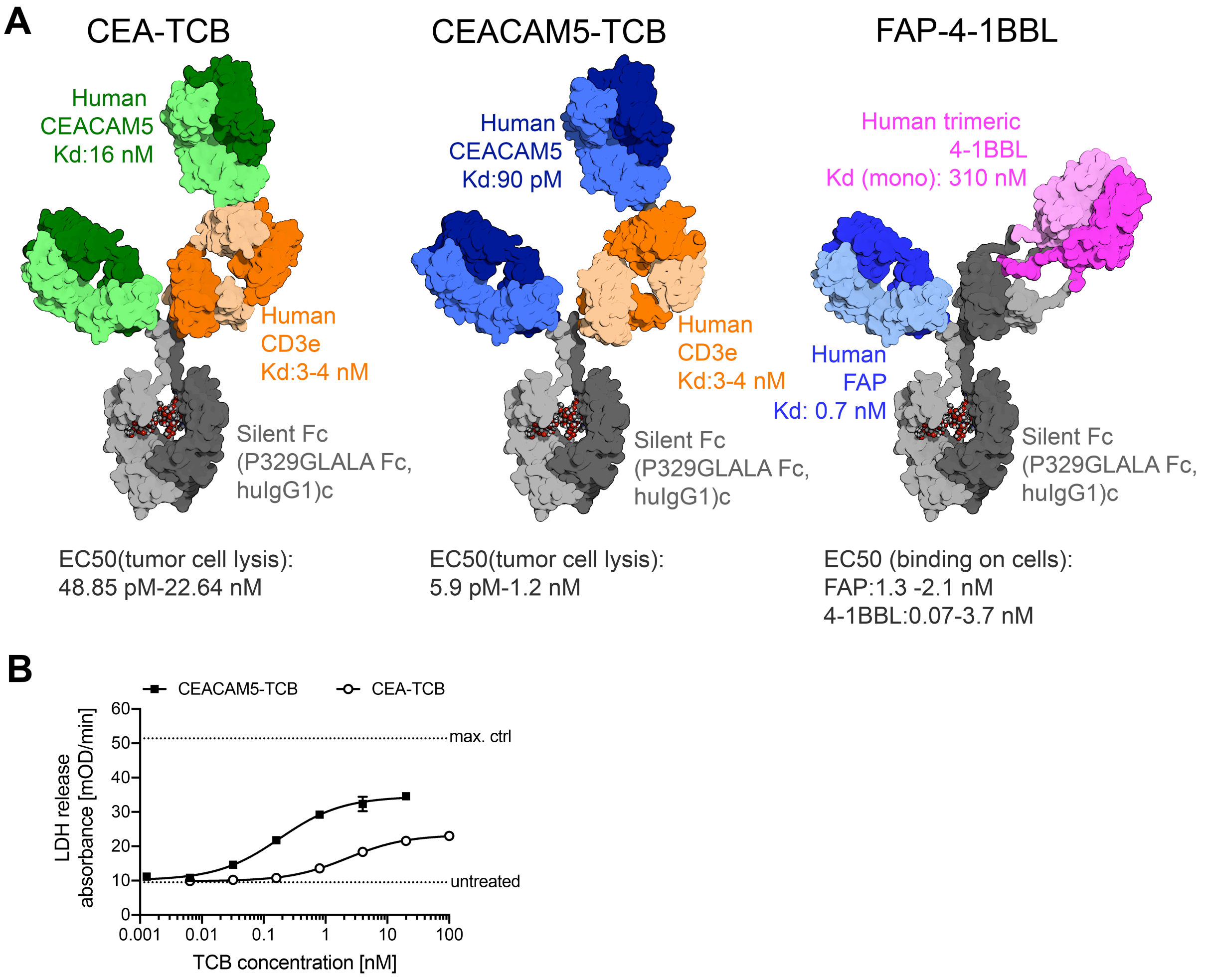

Figure S1. Description of CEA-TCB, CEACM5-TCB and FAP-4-1BBL constructs. (A) Three-dimensional structure, relative affinities for protein ligands of the different domains and EC50 concentrations as inferred from bioassays of the three bispecific protein constructs used in this manuscript. (B) LS800 colon cancer cell line concentration dependent cytotoxicity as induced by PBMCs treated with both CEA-TCB and CEACM5-TCB T-cell engagers as measured by lactate dehydrogenase (LDH) release.

Figure S2

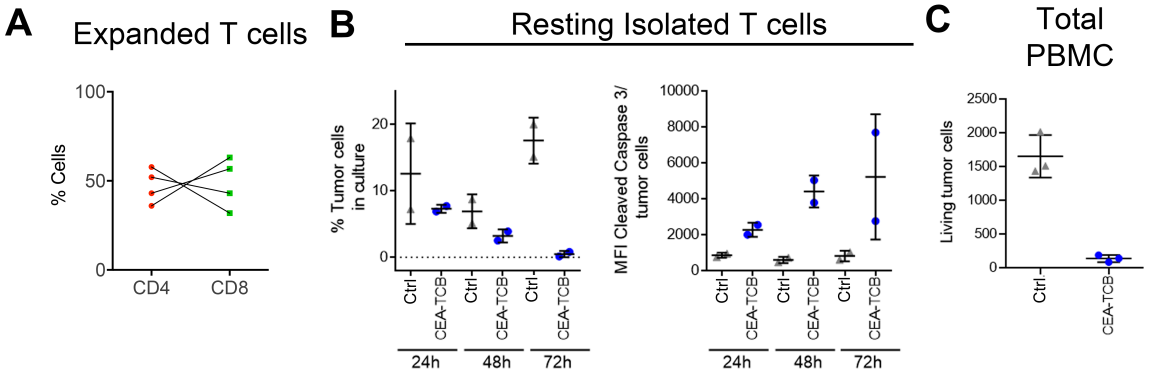

Figure S2. Non-activated T cells and PBMCs in 3D cytotoxicity experiments. (A) Relative percentage of CD4 and CD8 T cells in expanded T cell cultures used in cytotoxicity experiments against 3D tumor cell cultures. Four representative T-cell cultures are shown. (B) Cytotoxicity of resting (non-expanded) T cells isolated from healthy donors against 3D LS174T spheroids elicited by CEA-TCB and shown as percentage of tumor cells in culture and cleaved caspase 3 as assessed by flow cytometry at different time points. (C) Cytotoxicity of total PBMCs isolated from healthy donors against 3D LS174T spheroids elicited by CEA-TCB shown as percentage of tumor cells in culture after 48 h of co-culture assessed by flow cytometry.

Means ±SD are shown.

Figure S3

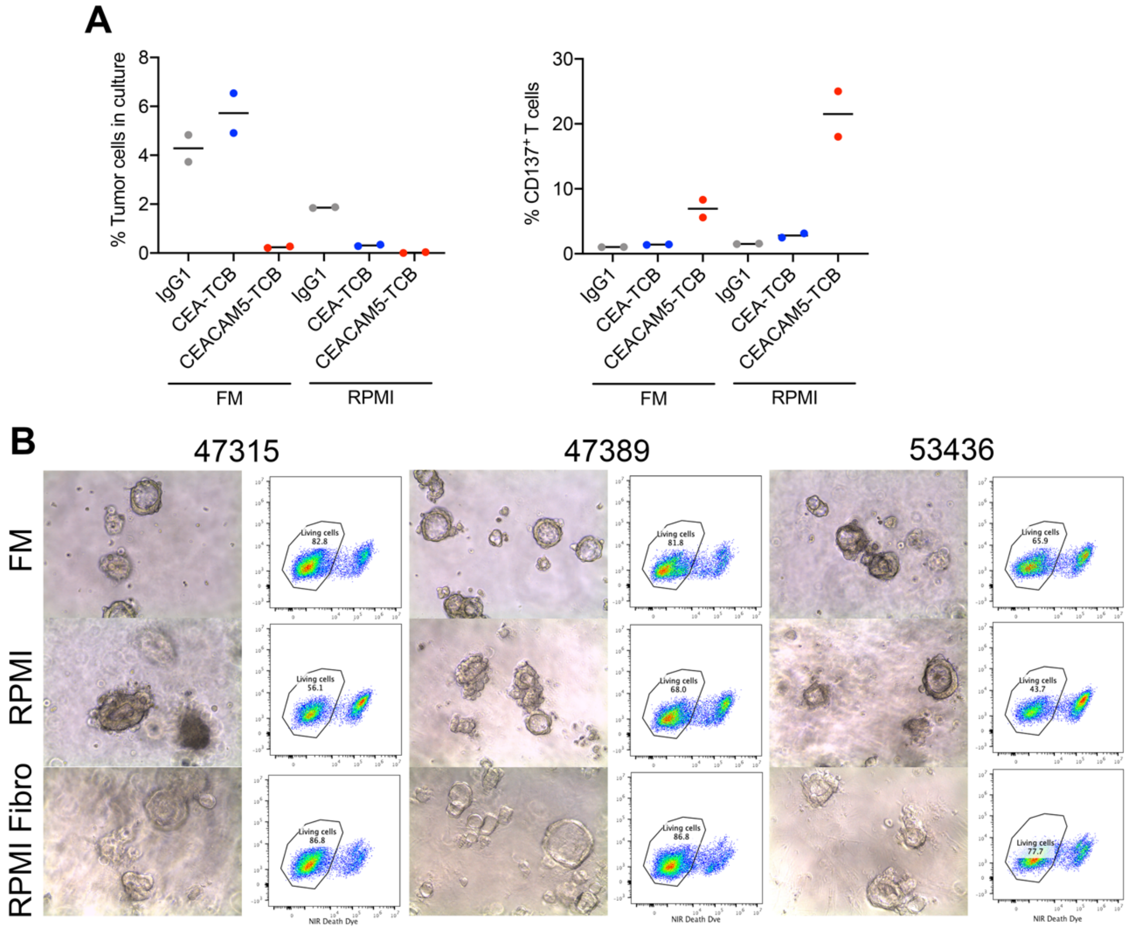

Figure S3. T-cell activation as induced by T-cell engagers and tumor organoid viability in RPMI versus organoid feeding media. (A) Activated T cells were cocultured with 47389 tumor derived organoids in the presence of CEA-TCB and CEACM5-TCB T cell engagers and recovered for flow cytometry analyses 24 hours later. Both the percentage of surviving tumor cells and the number of activated T cells in the coculture (measured as %CD137 positive T cells) are shown. (B) 72 h cultures of three different colon cancer organoids set up in standard organoid feeding media, RPMI 10% FBS or RPMI 10% FBS including 15.000 tumor associated fibroblasts (from patient 47389). Microscopy images show the morphology and a flow cytometry analysis reflects the viability of the recovered cells.

Figure S4

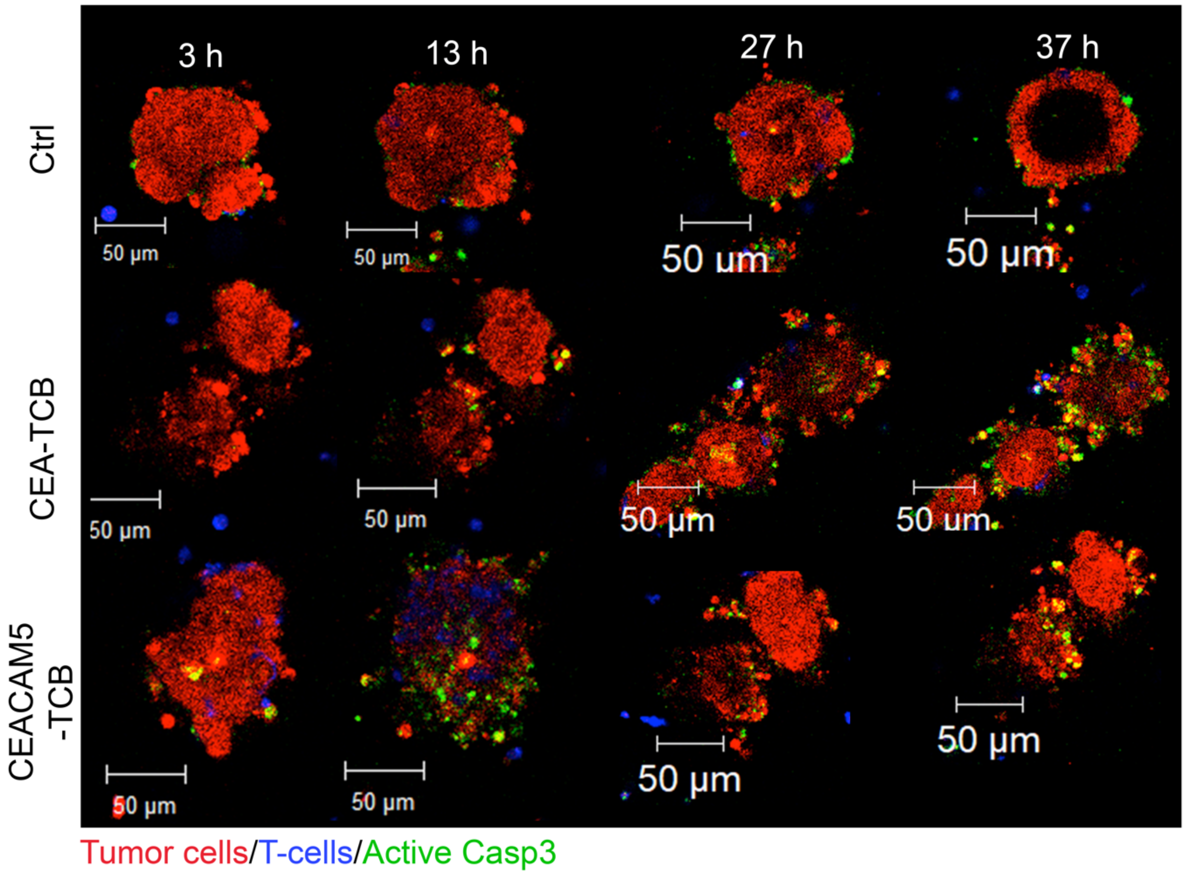

Figure S4. T-cell engagers induce cytotoxicity in primary colon cancer organoids using autologous expanded T cells. Organoids (Red) generated from colon cancer patients 47389 were co-cultured with T cells expanded from autologous PBMCs (Blue) and treated the CEA-TCB or CEACAM5-TCB T-cell engagers or an irrelevant mouse IgG control (Ctrl). Active caspase 3 is shown by a fluorescent probe (Green) that indicates apoptotic tumor cells. Representative confocal microscopy snapshots of time lapse videos of co-cultures treated with bispecific T-cell engagers.

Figure S5

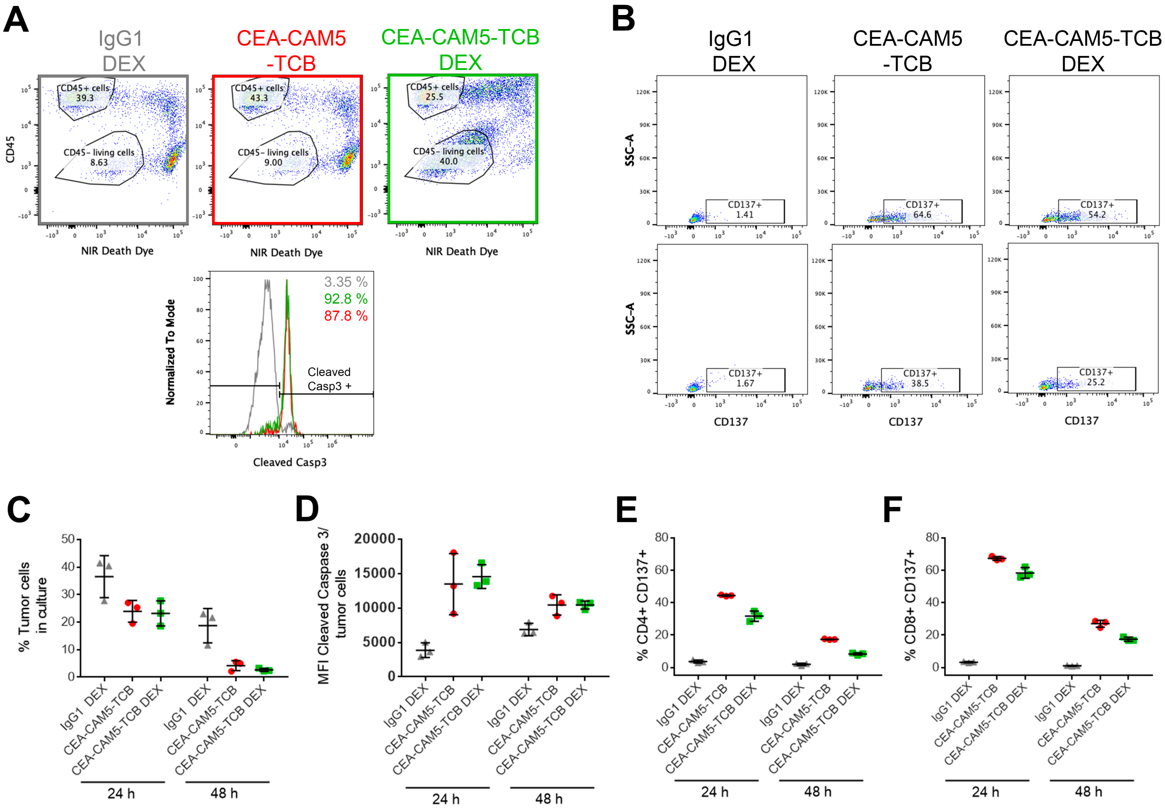

Figure S5. Corticosteroids do not impair CEA-TCB induced cytotoxicity. (A, C and D) Cytotoxicity of T cells against 3D LS174T-derived spheroids elicited by CEACAM5-TCB in the presence/absence of dexamethasone (5 μM, DEX) shown as percentage of surviving tumor cells in culture (A,C) and cleaved caspase 3 in tumor cells (A,D), that were assessed by flow cytometry at the indicated time-points. Panel (A) shows representative dot plots and histograms and (B and C) summary data. (B,E and F) Activation of T cells measured as surface CD137 induction on CD4 (B and E) and CD8 (B and F) T cells measured by flow cytometry in co-cultures. Panel (B) shows representative dot plots and histograms and (E and F) summary data. Representative experiment out of two rendering similar results. Means ±SD are shown.

Figure S6

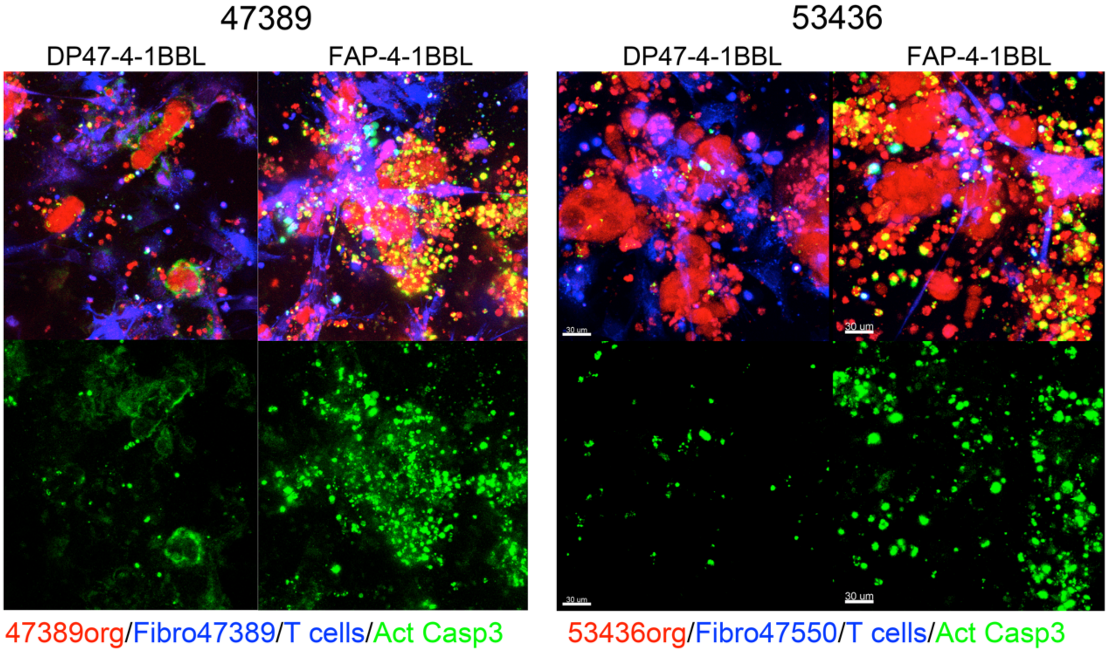

Figure S6. FAP-4-1BBL mediated co-stimulated cytotoxicity against 47389 and 53436 tumor organoids in cocultures with near-infrared-fluorescent tumor associated fibroblasts. Representative microscopy images of cocultures of tumor organoids 47389 (A) and 52436 (B)(Red), tumor associated fibroblasts (blue) and T cells (blue) treated with CEA-TCB plus UT-4-1BBL or FAP-4-1BBL following 48 hours of coculture. Induction of apoptosis in tumor cells is shown by the caspase 3 activity probe also in green. The green channel of the images is shown apart to better identify caspase 3 positive cells and differentiate them from green fibroblasts.

SUPPLEMENTARY VIDEO LEGENDS

Video S1. Time lapse microscopy of 3D tumor spheroids/T-cell co-cultures treated with CD3-CEA bispecific T-cell engagers. Time lapse microscopy videos of co-cultures of LS174T or HT29 spheroids (Red) with T cells (Blue) treated with CEA-TCB, CEACAM5-TCB T-cell engagers and an irrelevant mouse IgG_1 control (Ctrl). Active caspase 3 is shown by a fluorescent probe (Green) to indicate tumor cells entering apoptosis.

Video S2. Time lapse microscopy of colon cancer organoids/T-cell co-cultures treated with CD3-CEA bispecific T-cell engagers. Time lapse microscopy videos of co-cultures of 47389 and 53436 colon cancer organoids (Red) with T cells (Blue) treated with CEA-TCB, CEACAM5-TCB T-cell engagers and an irrelevant mouse IgG_1 control (Ctrl). Active caspase 3 is shown by a fluorescent probe (Green) to indicate tumor cells entering apoptosis.

Video S3. Time lapse microscopy of co-cultures of CEA^{Hi}, CEA^{Lo} spheroids and organoids with T cells treated with CD3-CEA bispecific T-cell engagers. The first sequence shows time lapse microscopy videos of co-cultures of HT29 (CEA^{lo}) and LS174T (CEA^{HI}) spheroids and T cells treated with the CEA-TCB T-cell engager. The second sequence shows time lapse microscopy videos of co-cultures of 47550 (CEA^{lo}) and 47389 (CEA^{HI}) colon cancer organoids and T cells treated with CEA-TCB T-cell engager. Active caspase 3 is shown by a fluorescent probe (Green) to indicate tumor cells entering apoptosis.

Video S4. Time lapse microscopy of co-cultures of primary colon cancer organoids with autologous fibroblasts and T cells. Time lapse microscopy videos of co-cultures of 47389 colon cancer organoids (Red) and 47389 tumor-associated fibroblasts (green) with T cells (Blue).

Video S4. Time lapse microscopy of co-cultures of primary colon cancer organoids with autologous fibroblasts and T cells treated with CEA T-cell

engagers plus FAP-4-1BBL. Time lapse microscopy videos of co-cultures of 47389 colon cancer organoids (Red) and 47389 tumor associated fibroblasts (green) with T cells (Blue) treated with 0.5 μg/ml CEA-TCB plus 10 μg/ml of UT-4-1BBL or FAP-4-1BBL. Active caspase 3 is shown by a fluorescent probe (Green) to indicate tumor cells entering apoptosis.
